# Canonical and Non-Canonical Functions of the Autophagy Machinery in MHC Restricted Antigen Presentation

**DOI:** 10.3389/fimmu.2022.868888

**Published:** 2022-03-04

**Authors:** Christian Münz

**Affiliations:** Viral Immunobiology, Institute of Experimental Immunology, University of Zürich, Zürich, Switzerland

**Keywords:** LC3-associated phagocytosis, unconventional secretion, extracellular vesicles, T cells, macroautophagy

## Abstract

Macroautophagy delivers cytoplasmic constituents for lysosomal degradation. Since major histocompatibility complex (MHC) class II molecules sample peptides after lysosomal degradation for presentation to CD4^+^ T cells, it was originally described that these peptides can also originate from macroautophagy substrates. In recent years it has become clear that in addition to this canonical function of the macroautophagy machinery during MHC class II restricted antigen presentation at least parts of this machinery are also used to regulate phagocytosis of antigens, degradation of MHC class I molecules, and unconventional secretion of antigens in extracellular vesicles, including virus particles. This review discusses how both canonical and non-canonical functions of the macroautophagy machinery influence antigen presentation on MHC class I and II molecules to CD8^+^ and CD4^+^ T cells. A better understanding of the molecular mechanisms by which the macroautophagy machinery is distributed between its canonical and non-canonical functions should allow targeting of antigens to these different pathways to influence MHC restricted presentation during vaccination against infectious diseases and tumors.

## Introduction on Autophagy

Autophagy, or self-eating, summarizes several pathways by which a cell can degrade its constituents in lysosomes (1–3). This group of pathways includes macroautophagy, microautophagy and chaperone-mediated autophagy, These pathways target cytosolic protein aggregates, cell organelles, intracellular pathogens and even surface receptors for degradation. In recent years it has become clear that the macroautophagy machinery is also used for non-canonical membrane trafficking events. The role of both canonical and non-canonical functions of the macroautophagy machinery for antigen processing and presentation to the immune system will be discussed in this review.

During macroautophagy its machinery of more than 40 autophagy related (ATG) proteins can be subdivided in different complexes (1, 2). A first protein kinase complex with ULK1 at its core and including ATG13 and FIP200 integrates signals of nutrient starvation to increase autophagy. For this purpose, depletion of ATP and an associated increase in AMP stimulates the AMP-activated protein kinase (AMPK) that in turn increases ULK1 function. Vice versa, nutrient availability stimulates the mechanistic target of rapamycin (mTOR) complex that inhibits ULK1 by phosphorylation. ULK1 modulates macroautophagy by phosphorylating many components of the macroautophagy machinery, including the type III phosphatidylinositol 3-kinase (PI3K) complex with VPS34 at its core and Beclin-1 and ATG14 as additional components (4). This complex phosphorylates membranes to which ubiquitin-like molecules will be covalently coupled. While yeast contains with ATG8 only one of these, human cells have six orthologues (LC3A, LC3B, LC3C, GABARAP, GABARAP-L1 and GABARAP-L2). These molecules are processed from their pro-forms by the cysteine proteases of the ATG4 family (ATG4A, ATG4B, ATG4C and ATG4D in humans). They then can be coupled to phosphatidylethanolamine or phosphatidylserine in membranes, converting for example LC3B-I into lipidated LC3B-II (5). This is performed by a ligase composed of ATG16L1, ATG5 and ATG12. ATG12 is another ubiquitin-like molecule that is covalently conjugated to ATG5 by ATG10 after activation by ATG7. Similarly, the ATG8 family is activated by ATG7 and conjugated by ATG3 before it is ligated to membranes *via* ATG16L1/ATG5/ATG12. The ATG8 ligase complex is recruited to these membranes by ATG16L1 binding to WIPI proteins that themselves are recruited to PI3P labelled membranes, generated by the VPS34 complex. ATG2 stabilized membrane channels and ATG9 containing membrane vesicles extend the ATG8 coupled membranes to the isolation membrane or phagophore that forms around macroautophagy substrates (6, 7). These substrates are linked to ATG8 or its human orthologues in the emerging isolation membrane by autophagy receptors, including p62, optineurin, NBR1, NDP52 and TAX1BP1 (8, 9). Once completed and engulfing the cargo as a double-membrane surrounded vesicle, this autophagosome travels along microtubules to fuse with late endosomes or lysosomes. Most ATG8 molecules are recycled from the outer autophagosomal membrane by ATG4 proteases (10). Fusion with late endosomes and lysosomes is then mediated by a machinery that includes syntaxin 17 and YKT6 (11, 12). The resulting autolysosome then degrades cargo and inner membrane of the fused autophagosome. Therefore, turnover of ATG8-II or its human orthologues including LC3B-II of the inner autophagosomal membrane, as well as degradation of the autophagy receptors such as p62 can be used to monitor flux through the macroautophagy pathway (13). The degradation products such as amino acids are exported from the autolysosome and thereby recycled by the cell. Therefore, macroautophagy delivers cellular components to lysosomal degradation which is monitored by the immune system for the presence of pathogen derived peptide sequences by MHC class II restricted antigen presentation.

## Intracellular Antigen Processing by Macroautophagy

**Figure 1:**
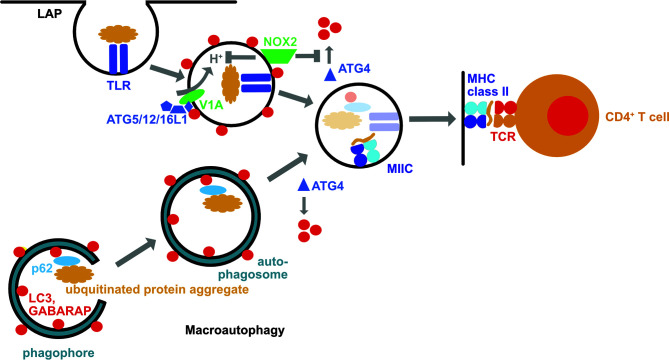
The macroautophagy machinery supports both intra- and extracellular antigen processing for MHC class II restricted presentation to CD4^+^ T cells. During LC3 associated phagocytosis (LAP) the cargo engages certain receptors, such as toll-like receptors (TLRs), including TLR2. This leads to the recruitment of the LC3 lipidation machinery (ATG5/12/16L1) *via* the vacuolar ATPase subunit V1A. LC3 lipidation is stabilized by the NADPH oxidase 2 (NOX2) which inhibits LC3 deconjugation by oxidation of ATG4. LC3 associated phagosomes eventually fuse with MHC class II containing compartments (MIIC). In these their cargo is degraded and loaded onto MHC class II molecules which are then transported to the cell surface for CD4^+^ T cell stimulation. Macroautophagy delivers intracellular antigens, such as ubiquitinated protein aggregates that are recruited into phagophores by autophagy receptors such as p62, with autophagosomes to MIICs. These intracellular antigens can then also be broken down by lysosomal hydrolases for loading onto MHC class II molecules.

Major histocompatibility complex (MHC) class II molecules present peptides of around 15 amino acids with a core binding sequence of 9 amino acids to helper CD4^+^ T cells (14). They sample these peptides from lysosomal degradation products in late endosomal MHC class II compartments (MIICs) (**Figure 1**). MHC class II molecules reach MIICs complexed to the chaperone invariant chain (Ii) which is then also degraded in MIICs. HLA-DM (H2-M in mice) then exchanges the last Ii remnant for peptides that bind with high affinity to the respective MHC class II molecules before these are displayed on the cell surface to CD4^+^ T cells.

Macroautophagy and chaperone-mediated autophagy can deliver antigens to MIICs for loading onto MHC class II molecules and CD4^+^ T cells stimulation (15–19) (**Figure 1**). These pathways allow sampling of both cytosolic and nuclear antigens (20–22). MHC class II ligands include also ATG8 orthologues, like LC3B, GABARAP and GABARAP-L1, as well as autophagy receptors, such as TAX1BP1 (19, 23). This pathway might be especially important in cell types with limited phagocytic activity such as B, epithelial and endothelial cells. Along these lines, nuclear antigen 1 (EBNA1) of the Epstein Barr virus (EBV) was found to be processed *via* macroautophagy in EBV transformed B cells for presentation on MHC class II molecules to CD4^+^ T cells (16, 20). Indeed, one of the peptidomes that identified ATG8 orthologues and autophagy receptors as sources of MHC class II ligands was eluted from EBV transformed B cells (19). EBV activates these B cells with its oncogene latent membrane protein 1 (LMP1), stimulating macroautophagy (24). Such LMP1 activated B cells have recently been shown to efficiently stimulate CD4^+^ T cells against tumor associated antigens (TAAs) (25). In this study, LMP1 expression resulted in TAA expression and these TAAs were then intracellularly processed onto MHC class II molecules. Such LMP1 expressing B cells were capable to stimulate TAA specific cytotoxic CD4^+^ T cells and even prime such CD4^+^ T cell specificities from tumor patients. These studies suggest that B cells might utilize autophagic pathways to load their MHC class II molecules and after proper activation of these antigen presenting cells, including by EBV infection or LMP1 expression, allows them to potently stimulate even cytotoxic CD4^+^ T cell responses.

Additional cell types that might depend on autophagic pathways to load their MHC class II molecules are epithelial and endothelial cells. In particular, thymic epithelial cells that are involved in T cell education and central tolerance have high constitutive macroautophagy (26). Autophagosomes fuse in these cells with MIICs (27). ATG5 deficient thymic epithelial cells present an altered peptide repertoire on their MHC class II molecules (26). This results in changed positive and negative CD4^+^ T cell selection in the thymus, diminishing positive selection of some T cell receptor specificities and resulting in an autoreactive T cell repertoire. Targeting antigens for macroautophagy in medullary thymic epithelial cells *via* fusion with LC3B, a strategy that has been used to increase MHC class II presentation for a variety of viral and tumor antigens (17, 28–31), leads to negative selection of CD4^+^ T cells that are specific for the respective antigens (32). In addition to this role in central tolerance, endogenous antigen processing *via* macroautophagy might also be important for peripheral tolerance and regulatory CD4^+^ T cell stimulation. Along these lines lymphatic endothelial cells express MHC class II molecules to stimulate regulatory CD4^+^ T cells (33–36). Autophagosomes fuse with MIICs in these cells (36). Loss of ATG5 compromises regulatory T cell proliferation and phenotype, and results in exacerbated collagen induced arthritis with Th17 infiltrations (36). The macroautophagic machinery is then required in dendritic cells to maintain regulatory CD4^+^ T cell function (37). Vice versa, regulatory CD4^+^ T cells down-modulate the macroautophagy machinery in dendritic cells to prevent autoimmunity (38). Therefore, macroautophagic self-antigen processing for MHC class II presentation in lymphatic endothelial cells and the macroautophagy machinery in dendritic cells might sustain regulatory CD4^+^ T cell function in tissues to prevent autoimmunity.

## Extracellular Antigen Processing by LC3-Associated Phagocytosis

In addition to this canonical function of the macroautophagy machinery for intracellular antigen presentation on MHC class II molecules, components of this machinery support also extracellular antigen presentation to CD4^+^ T cells (39–46). Revealing characteristics of this non-canonical function of the macroautophagy machinery, it was already noted in yeast deficient for ATG4 and transgenic for the processed ATG8-I, allowing therefore conjugation but not deconjugation of ATG8 at membranes, that ATG8 can be coupled to endosomal, vacuolar and endoplasmic reticulum membranes (47). Along these lines it has been shown that ATG8 orthologues, including LC3B, can also be conjugated to the cytosolic side of phagosomes during LC3-associated phagocytosis (LAP) (41, 48) (**Figure 1**). In contrast to macroautophagy, the ULK1 complex is not required for LAP, but ATG16L1 is recruited to phagosomes *via* its WD40 domain, and assembly of the NADPH oxidase 2 (NOX2) at this membrane as well as its function is required for LAP (41, 44, 49). NOX2 assembly is facilitated by the engagement of pathogen associated molecular pattern (PAMP) receptors during LAP cargo uptake. Along these lines engagement of toll-like receptor 2 (TLR2), of the C-type lectin Dectin-1, of Fc receptors and of the phosphatidylserine receptor TIM4 have been described to stimulate LAP (40, 41, 48, 50). The function of NOX2 during LAP could at least be twofold. NOX2 has been reported to neutralize the pH of maturing phagosomes *via* its reactive oxygen species (ROS) production (51), and pH neutralization during endosome maturation seems to accumulate components of the vesicular ATPase at these vesicles that then recruits ATG16L1 *via* its WD40 domain (52) (**Figure 1**). The ATG16L1, ATG5 and ATG12 dependent conjugation of LC3B at membranes of such endosomes with compromised acidification seems to preferentially occur to phosphatidylserine (5). A second function of NOX2 produced ROS is the regulation of LC3B deconjugation by ATG4B from phagosomal membranes (46) (**Figure 1**). It has been shown that oxidation inhibits ATG4 activity, and even leads to its aggregation (46, 53–55). Accordingly, phagosomes with the highest ROS production sustained LC3B conjugation, and presented with delayed acidification and lysosome fusion (41, 46, 51). ROS insensitive ATG4B was able to deconjugate LC3B and inhibit LAP (46). Even so these molecular features of NOX2 and LC3B associated phagosomes are most consistent with delayed endocytosed cargo degradation, as has been observed for human myeloid cells (41, 46), LAP supports more rapid endocytosed pathogen degradation in mouse myeloid cells (48–50, 56). In the absence of efficient lysosomal fusion LAP directs phagosomes to other endosomal compartments, such as TLR containing endosomes, as observed in plasmacytoid dendritic cells and lung epithelial cells (57, 58).

Alternatively, LAP directs its cargo also for efficient MHC class II restricted antigen presentation (39–46). This has been demonstrated for bacterial, yeast, model, tumor, and autoantigens. Diminished CD4^+^ T cell stimulation in the absence of LAP supported MHC class II restricted antigen presentation by dendritic cells leads also to an amelioration of experimental autoimmune encephalomyelitis (EAE), an autoimmune CNS disease in mice, after adoptive transfer of autoimmune CD4^+^ T cells into mice that are either deficient in ATG5 or NOX2 (43, 59, 60). However, LAP in microglia, the resident macrophages of the brain, does not seem to be required for this LAP dependent autoantigen presentation to elicit EAE after autoimmune CD4^+^ T cell transfer (61). In addition, a LAP-like pathway supports bacterial outer membrane vesicle (OMV) processing for the stimulation of regulatory CD4^+^ T cells in the mouse gut (42). Expression of ATG4B that is insensitive to NOX2 produced ROS inhibits endocytosed yeast antigen presentation on MHC class II molecules by human macrophages to *Candida albicans* specific CD4^+^ T cells (46). Therefore, the non-canonical role of the macroautophagy machinery during phagocytosis (LAP) redirects extracellular antigens for more efficient antigen presentation on MHC class II molecules to CD4^+^ T cells.

## Attenuation of MHC Class I Restricted Antigen Presentation by the Macroautophagy Machinery

**Figure 2:**
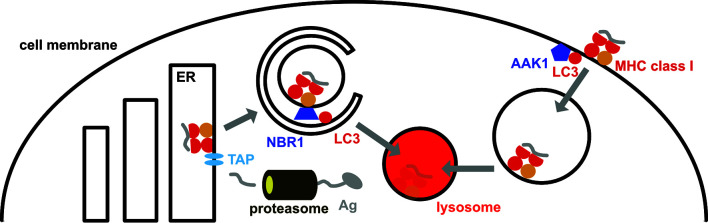
MHC class I surface expression is down-regulated by the macroautophagy machinery. MHC class I molecules are loaded in the endoplasmic reticulum (ER), primarily with peptides that originate from proteasomal degradation and are then imported into the ER *via* the transporter associated with antigen presentation (TAP). On their way to the cell membrane from the ER, MHC class I molecules are targeted for macroautophagy by the autophagy receptor NBR1, resulting in their lysosomal degradation in pancreatic ductal adenocarcinoma (PDAC) cells. In addition, LC3 lipidation recruits parts of the internalization machinery to MHC class I molecules at the cell membrane, including AP2 associated kinase 1 (AAK1). This leads to a more rapid internalization and degradation of MHC class I molecules in dendritic cells.

In contrast to MHC class II ligands, peptides that are presented by MHC class I molecules originate primarily from proteasomal degradation in cytosol and nucleus (62) (**Figure 2**). The peptides are then translocated by the transporter associated with antigen presentation (TAP) into the endoplasmic reticulum (ER) where they get loaded onto MHC class I molecules which is co-translationally inserted into the ER. Once a high affinity octa- or nonameric peptide has been inserted into the peptide binding groove, the MHC class I loading complex disassembles and the MHC class I plus peptide complex is transported to the cell surface for surveillance by CD8^+^ T cells.

In mice with compromised macroautophagy in dendritic cells (ATG5, ATG7 or ATG16L1 deficiency) hyperreactive CD8^+^ T cell responses were noted (63, 64). These result in part from stabilized MHC class I molecules on the cell surface of dendritic cells, resulting in their accumulation and increased CD8^+^ T cell stimulation (63). This MHC class I accumulation was due to decreased internalization (**Figure 2**). LC3 lipidation was required for recruitment of parts of the MHC class I internalization machinery to MHC class I molecules, including AP2 associated kinase 1 (AAK1) (63). In the absence of AAK1 mediated MHC class I internalization dendritic cells stimulated stronger influenza A virus specific CD8^+^ T cell responses *in vitro* and *in vivo*. These inversely correlated with influenza A viral loads after infection. Similarly, higher lymphocytic choriomeningitis virus (LCMV) specific immune responses were detected in the absence of LC3 lipidation. In addition, the macroautophagy machinery supports degradation of extracellular antigen storage compartments in dendritic cells and thereby also compromises MHC class I restricted antigen cross-presentation (65). Enhanced internalization by LC3 lipidation extends also to the non-classical MHC class I molecule CD1d (66). CD1d presents glycolipids to invariant NKT cells. In the absence of ATG5 of dendritic cells CD1d levels are elevated on dendritic cells and NKT cell responses are more efficiently stimulated *in vitro* and *in vivo*. This leads to attenuated infection with the NKT cell sensitive bacterial pathogen *Sphingomonas paucimobilis* in mice. Thus, the LC3 lipidation complex negatively regulates surface expression of classical and non-classical MHC class I molecules on dendritic cells, resulting in diminished CD8^+^ T and NKT cell stimulation.

Another cell type in which the autophagy machinery down-regulates MHC class I molecules are pancreatic ductal adenocarcinoma (PDAC) cells (67, 68). PDAC cells express low levels of surface MHC class I molecules which are diverted for lysosomal degradation by NBR1 dependent macroautophagy (67) (**Figure 2**). Macroautophagy inhibition by ATG3 down-regulation or lysosomal degradation attenuation restores MHC class I surface expression. Together with immune check-point blockade treatment by antibody blocking of PD-1 and CTLA-4, macroautophagy inhibition allows for efficient CD8^+^ T cell stimulation in PDAC tumors and decreased pancreatic cancer growth in mice. This macroautophagy dependent degradation of MHC class I molecules in PDAC cells seems to be stimulated by autocrine progranulin secretion of the cancer cells (68). Antibody mediated blocking of progranulin restores MHC class I expression on PDAC cells and leads to attenuated pancreatic cancer growth in mice. Thus, the macroautophagy machinery targets MHC class I molecules for lysosomal degradation in PDAC cells, allowing these to escape CD8^+^ T cell mediated immune control.

## Antigen Secretion With the Assistance of the Macroautophagy Machinery

In addition to influencing the trafficking of MHC class I molecules in dendritic and tumor cells, the autophagy machinery has also been implicated in releasing antigens for optimal cross-presentation on MHC class I molecules (69, 70). Release of extracellular vesicles that carry antigens for efficient cross-presentation was found to be enhanced by inhibition of lysosomal degradation (71, 72). Furthermore, it was noted that these extracellular vesicles can be efficiently processed for antigen presentation on MHC class I and II molecules (73, 74). Inhibition of lysosomal acidification or of the fusion machinery between autophagosomes and lysosomes leads to the release of vesicles that contain autophagy receptors, such as p62 and NBR1 (75, 76). These receptors recruit most likely ubiquitinated protein aggregates of both long- and short-lived proteins (71) into autophagosomes whose inner membrane is then released for unconventional secretion after autophagosome fusion with multivesicular bodies (MVBs) and successive exocytosis from MVBs. This pathway of antigen transfer that can be enhanced upon inhibition of autophagosome degradation in lysosomes is most likely also used by physiological substrates, such as acyl coenzyme A-binding protein (ACBP) in yeast and IL-1β in mammalian cells (77–80). Furthermore, it might be related to secretory lysosomes (81, 82). Thus, the canonical functions of the macroautophagy machinery might select ubiquitinated antigens with autophagy receptors into autophagosomes which fuse with MVBs, followed by exocytosis of the inner autophagosomal membrane and its content to become extracellular vesicles. The contained antigens are efficiently presented on MHC class I and II molecules of bystander antigen presenting cells.

In addition to this secretion of inner autophagosomal membranes, the macroautophagy machinery supports direct recruitment of LC3 binding proteins at the limiting membrane of MVBs for unconventional secretion in their intravesicular bodies (83). This pathway targets RNA binding proteins (RBPs), such as heterogeneous nuclear ribonucleoprotein K (HNRNPK) and scaffold-attachment factor B (SAFB), by direct interaction with LC3 at the limiting MVB membrane that then invaginates to become an intravesicular body containing RBPs and their RNA. This membrane invagination requires neutral sphingomyelinase 2 (nSMase2), and the factor associated with nSMase2 activity (FAN) that directly interacts with LC3. Thus, this second unconventional secretion pathway that uses the LC3 conjugation machinery delivers LC3 interacting RBPs for release in extracellular vesicles.

Viruses have been described to carry lipidated ATG8 orthologues, including LC3B-II, in their envelope or pseudoenvelope (84–88). Indeed, inhibition of the LC3 conjugation machinery, including of ATG5, ATG12 and ATG16L1, inhibit the non-lytic release of both herpes and picornaviruses. Which of the two above outlined unconventional secretion pathways these viruses, however, use to acquire LC3 coupled membranes remains unclear because the role of autophagy receptors versus viral proteins that might directly interact with LC3 in this process have not been elucidated. Thus, the macroautophagy machinery supports at least two different pathways of unconventional secretion in extracellular vesicles. These are used to efficiently transfer antigens for MHC class I and II restricted presentation, but are also hijacked by pathogens, mainly viruses, for their release.

## Conclusions and Future Directions

In recent years it was realized that parts of the macroautophagy machinery are used in modular form for additional membrane trafficking events, including antigen phagocytosis, surface receptor endocytosis, and exocytosis of extracellular vesicles. All of these additional pathways intersect with MHC class I and II restricted antigen presentation for CD8^+^ and CD4^+^ T cell stimulation, respectively. While for some of these pathways, like LAP, molecular interactors that recruit only parts of the macroautophagy machinery, such as ATG16L1 interaction with the vesicular ATPase and NOX2 regulation of ATG4, are starting to be defined (46, 52), additional information needs to be gathered on how the macroautophagy machinery is distributed between the different membrane trafficking events that it regulates.

Such detailed information seems necessary in order to separately influence the different pathways that are regulated by the macroautophagy machinery and uncouple its pro- and anti-immunogenic functions, e.g. avoiding MHC class I degradation but maintaining enhanced MHC class II antigen presentation during immune responses. Along these lines, antigen targeting *via* different autophagy receptors or different ATG8 orthologues could be further explored, as has been initially reported for LC3B fusion antigens (17, 28–31). Combined with specific targeting for intracellular expression of such targeted antigens in professional antigen presenting cells such as dendritic or B cells by suitable viral vectors or liposome encapsulated mRNA, such approaches could refine vaccination against infectious diseases and tumors.

## Author Contributions

The author confirms being the sole contributor of this work and has approved it for publication.

## Funding

Research in my laboratory is financially supported by Cancer Research Switzerland (KFS-4962-02-2020), HMZ ImmunoTargET of the University of Zurich, the Cancer Research Center Zurich, the Sobek Foundation, the Swiss Vaccine Research Institute, the Swiss MS Society (2021-09), Roche, Novartis, Innosuisse (52533.1), and the Swiss National Science Foundation (310030_204470/1, 310030L_197952/1 and CRSII5_180323). The funders were not involved in the writing of this article or the decision to submit it for publication.

## Conflict of Interest

The author declares that the research was conducted in the absence of any commercial or financial relationships that could be construed as a potential conflict of interest.

## Publisher’s Note

All claims expressed in this article are solely those of the authors and do not necessarily represent those of their affiliated organizations, or those of the publisher, the editors and the reviewers. Any product that may be evaluated in this article, or claim that may be made by its manufacturer, is not guaranteed or endorsed by the publisher.
